# Development and internal validation of a post-retrieval machine learning models for OHSS risk stratification in assisted reproductive technology: an exploratory study

**DOI:** 10.3389/fendo.2026.1826843

**Published:** 2026-05-25

**Authors:** Xiangqian Meng, Tian Xia, Si Wei, Qian Xu, Dongzhi Yuan, Linlin Yu

**Affiliations:** 1 Embryo Laboratory, Jinxin Xinan Women & Children's Hospital, Chengdu, Sichuan, China; 2 West China School of Basic Medical Sciences & Forensic Medicine, Sichuan University, Chengdu, Sichuan, China; 3 School of biomedical sciences, Hunan University, Changsha, Hunan, China; 4 Department of Physiology, West China School of Basic Medical Sciences and Forensic Sciences, Sichuan University, Chengdu, Sichuan, China; 5 Chengdu Women’s and Children’s Central Hospital, School of Medicine, University of Electronic Science and Technology of China, Sichuan, China

**Keywords:** area under the receiver operating characteristic curve (AUC), assisted reproductive technology (ART), controlled ovarian hyperstimulation (COH), machine learning (ML), ovarian hyperstimulation syndrome (OHSS)

## Abstract

**Objective:**

This study aimed to explore the feasibility of developing and internal validating machine learning (ML) models for post-retrieval ovarian hyperstimulation syndrome (OHSS) risk stratification in patients undergoing assisted reproductive technology (ART).

**Methods:**

In this exploratory, single-center, retrospective study, three ML models were established using clinical and laboratory data from infertile patients undergoing controlled ovarian hyperstimulation (COH) at Jinxin Xinan Women & Children Hospital between 2020 and 2023. The models were evaluated comprehensively using the area under the receiver operating characteristic curve (AUC), accuracy, sensitivity, specificity, F1 score, calibration curves, and decision curve analysis. Model interpretability was achieved using the SHapley Additive exPlanations (SHAP) method to identify key predictors and clarify the basis of model decisions.

**Results:**

A total of 500 infertility patients were enrolled, including 40 diagnosed with OHSS. Participants were randomly divided into a training set (n = 350) and a test set (n = 150). Among the three ML models developed, the RandomForest model demonstrated the best discriminatory performance in the test set, achieving an accuracy of 0.79, sensitivity of 0.75, specificity of 0.80, F1 score of 0.37, and an AUC of 0.81 (95% CI: 0.68-0.95). The Least Absolute Shrinkage and Selection Operator (LASSO) and SHAP analysis identified number of oocytes retrieved, age, P levels on the trigger day, and ratio of baseline LH/FSH as the most influential predictors in the model.

**Conclusions:**

This proof-of-concept study demonstrates that the ML-based approach shows exploratory promise for OHSS risk stratification in infertility patients undergoing ART. By integrating demographic and clinical variables, the model - particularly the Random Forest algorithm - may serve as hypothesis-generating framework for informing post-retrieval risk stratification, ultimately mitigating the risk of OHSS. Combining ML with SHAP provided explicit, individualized risk interpretation that may help clinicians intuitively understand the impact of key predictive features. However, this interpretability does not imply validated clinical utility. These findings are exploratory and hypothesis-generating. They require rigorous external validation in larger, multi-center prospective studies before clinical implementation.

## Introduction

Ovarian Hyperstimulation Syndrome (OHSS) remains a clinically significant complication of controlled ovarian hyperstimulation (COH) in assisted reproductive technology (ART) (1, 2). The overall incidence of mild OHSS ranges from 20% to 30%, while moderate-to-severe cases occur in 1% to 6% of high-risk populations (3–5). Clinical manifestations range from mild abdominal distension, nausea, and ovarian enlargement to severe sequelae including massive ascites, pleural effusion, and thromboembolic events (6, 7). This potentially life-threatening condition is pathologically characterized by systemic capillary hyperpermeability, leading to hemoconcentration and third-space fluid sequestration, which frequently necessitates intensive care admission in severe cases (8, 9). The syndrome not only affects patient’s health, but also results cycle cancellation, prolongs treatment, increases healthcare costs, and inflicts substantial psychological distress (10). Current clinical strategies for OHSS prediction primarily relies on clinicians’ experiential judgment and conventional biomarkers [e.g., serum Anti-Müllerian Hormone (AMH), antral follicle count (AFC)], patient history [e.g., previous OHSS, polycystic ovary syndrome (PCOS)], and stimulation response monitoring (11). However, the pathophysiological complexity of OHSS involves non-linear interactions between genetic predisposition, angiogenic factors (notably VEGF and IL-6), gonadotropin receptor polymorphisms, and iatrogenic hCG exposure, with emerging evidence implicating specific immune cell phenotypes, plasma metabolites, and novel circulating proteins (such as SBSN, CD244, ENO3, and TXNDC12) in its pathogenesis (12–16). Traditional statistical methods like univariate or multivariate logistic regression are often based on linear assumptions and limited variable combinations, struggling to capture non-linear relationships, complex interactions within clinical data, thereby restricting predictive accuracy and sensitivity, and failing to effectively identify all high-risk patients (17, 18).

The application of multiple machine learning (ML) models offers a transformative paradigm for enhancing OHSS prediction (19, 20). ML algorithms excel at identifying intricate, non-linear patterns within large, multidimensional datasets, surpassing the analytical capacity of conventional statistics (17, 18). The novelty of employing multiple ML models [e.g., Random Forest, eXtreme Gradient Boosting (Xgboost), etc.] has advantages (19, 20). The theoretical justification is robust, evidenced by ML’s proven superiority over traditional models in predicting complex medical outcomes across diverse fields (17, 20, 21). However, ML models are often perceived as “black boxes”, with opaque decision-making processes that pose significant barriers to clinical implementation. SHapley Additive exPlanations (SHAP), a game theory-based approach, addresses this limitation by quantifying the contribution of each feature to individual predictions, thereby providing transparent, patient-level risk explanations (22, 23). The integration of high-performance ML models with SHAP interpretability represents a promising strategy for developing clinically actionable risk stratification tools.

Therefore, we designed a comprehensive study employing three distinct ML models to predict OHSS risk in infertile patients undergoing COH based solely on readily available baseline clinical indicators, and to achieve model interpretation through SHAP methodology. This multi-model strategy aims to identify key predictive factors and provide clinicians with scientific evidence to support post-retrieval risk stratification for timely preventive interventions to mitigate OHSS-related morbidity.

## Materials and methods

### Study population

A retrospective cohort study was conducted at Jinxin Xinan Women & Children Hospital from January 2020 to December 2023. The study population comprised patients undergoing IVF or ICSI cycles who received either a single hCG trigger, a single GnRH agonist (GnRHa) trigger, or a dual trigger (hCG plus GnRHa) for COH. Participants were included when they demonstrated preserved ovarian reserve, defined by basal FSH ≤15 IU/L and E_2_ ≤60 pg/mL, with anatomically normal reproductive organs verified through hysterosalpingographic or hysteroscopic assessment. Participants were excluded if they presented a history of previous poor ovarian response, gonadotropin treatment within 3 months before the study, hydrosalpinx, ovarian endometrioma, or relevant uterine disorders (adenomyosis, congenital malformations, endometrial polyps, intrauterine adhesions), as well as parental chromosomal abnormalities. Cycles initially planned for IUI and later converted to IVF, and fertility preservation for malignancy were also disqualified.The study followed the TRIPOD guideline (24). Further details are available in **Supplementary Table 1**.

### Diagnostic criteria for OHSS

OHSS was diagnosed based on the 2024 American Society for Reproductive Medicine (ASRM) guideline on Prevention of moderate and severe OHSS: a guideline (18, 25).

### ML models overview

Classical statistical modelling was originally designed for datasets with a few dozen input variables and small to moderate sample sizes. As the number of variables per subject increases, statistical inferences become less precise. ML algorithms can effectively capture complex relationships in high-dimensional data, even when the data is gathered without a carefully controlled experimental design (26). These capabilities have demonstrated excellent outcomes across diverse disciplines (17, 26). In the present study, we developed a predictive model for OHSS using relevant variables. Key features associated with OHSS risk were identified through Least Absolute Shrinkage and Selection Operator (LASSO) logistic regression analysis. Subsequently, three ML algorithms: Logistic Regression (Logistic), Random Forest, and Xgboost, were trained and optimized using bootstrap resampling (1000 iterations). Model performance was assessed using multiple metrics, such as the area under the receiver operating characteristic curve (AUC-ROC), accuracy, sensitivity, specificity, precision, F1 score, calibration curves, and decision curve analysis.

### Feature selection pipeline and ML models establishment

**Figure 1:**
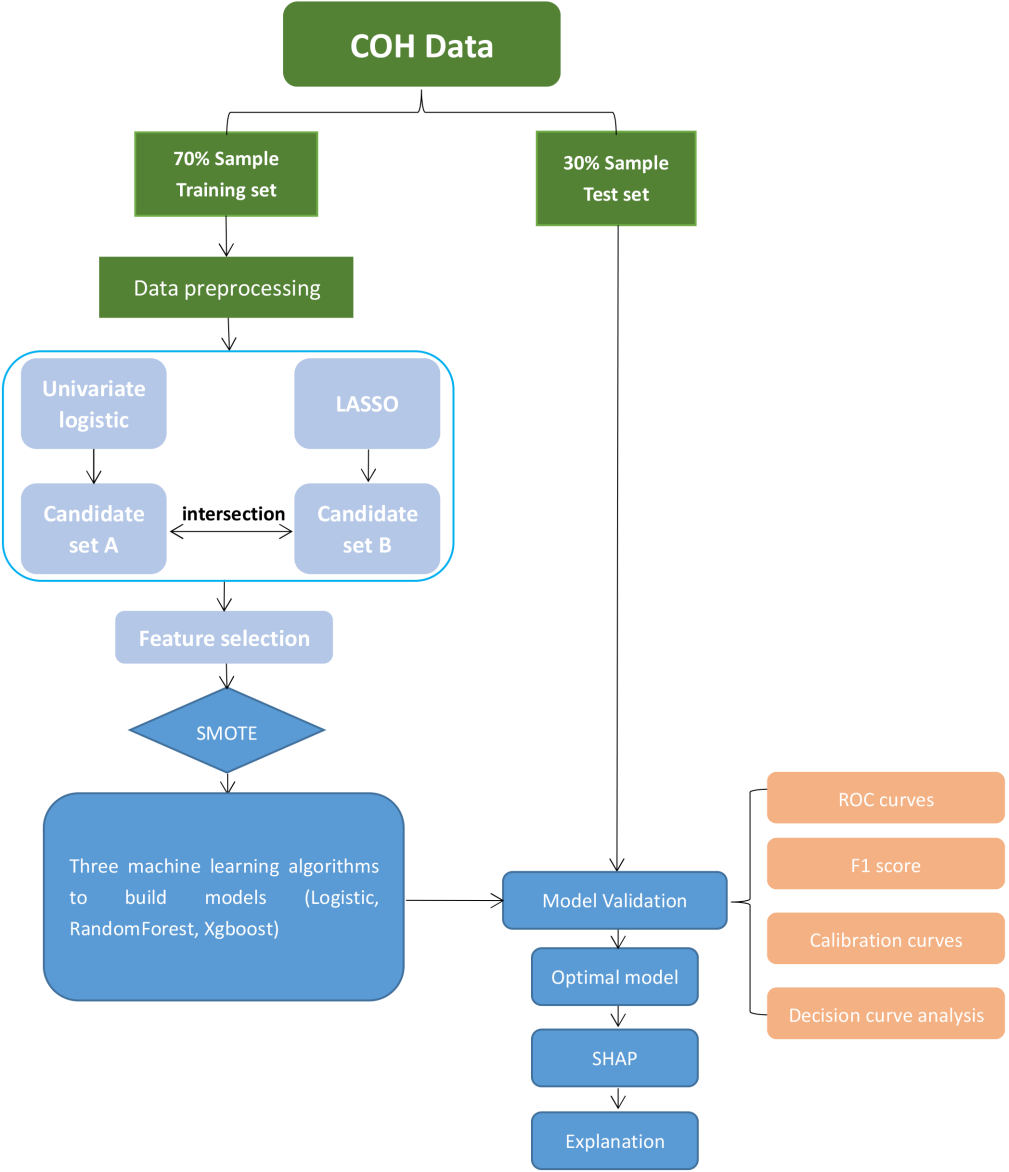
Analysis flow for the development and evaluation of models.

The dataset was first randomly splited into training and test sets in a 70%:30% ratio (**Figure 1**). Feature selection was conducted strictly within the training set using two parallel approaches. First, univariate logistic regression on the 25 variables identified features with P < 0.05 (candidate set A). Second, LASSO regression was applied to the original 25 variables, with the penalty parameter optimized via 10−fold inner cross−validation (minimum mean cross−validated deviance; λ), yielding non−zero coefficient features (candidate set B). The final set of predictors for model construction was defined as the intersection of set A and set B. All feature selection steps were completed before any evaluation on the validation set, ensuring no information leakage. The initial dataset exhibited a significant class imbalance. Hence, the training set was augmented with synthetic samples generated by Synthetic Minority Over-sampling Technique (SMOTE) to achieve a 1:1 ratio and enhance the ML model’s performance (27–29) (**Figure 1**). ML models were built on the training set. Bootstrap resampling with 1000 iterations was used to optimize the hyper-parameters and assess the stability of ML models. Finally, the performance of each model was evaluated and compared in the test set. Three ML models were trained with fixed random seeds to ensure reproducibility (**Figure 1**). Through comprehensive evaluation of multiple evaluation indicators (ROC curves, F1 score, Calibration curves and Decision curves analysis), the best performing model among the three models was selected for further risk stratification to distinguish between OHSS and non-OHSS (NOHSS) (**Figure 1**).

### Feature selection stability

The stability of predictor rankings was evaluated using bootstrap resampling (1000 iterations) from the test set. At each iteration, the Random Forest model was refitted on a bootstrap sample, and permutation-based feature importance was recorded. Selection frequency (proportion of resamples with ranking ≤ 3), mean rank, and rank standard deviation were computed for each feature. Stability was classified as High (selection frequency ≥ 0.8), Moderate (0.6 ≤ selection frequency < 0.8), or Low (selection frequency < 0.6).

### Interpretation of models and outcomes

Drawing on cooperative game theory, we employed SHAP to render the model’s outputs and optimization trajectory interpretable (22, 23) (**Figure 1**). SHAP values quantified each feature’s marginal contribution to the predictive outcome; the resulting swarm plot depicted the complete distribution of these contributions across observations, whereas case-level SHAP profiles elucidated how individual features influenced specific predictions. Recursive feature elimination was subsequently applied to retain only the most informative variables, yielding a parsimonious yet transparent model.

### Statistical methods

In the study, the Kolmogorov-Smirnov test was used for continuous variables. As all continuous variables were non-normally distributed, they are represented as median (IQR) and compared using the Mann-Whitney U test. Categorical data were presented as frequencies (proportions) and compared using the Pearson chi-square test. The ML models used all predictor variables as independent variables. All models were built with OHSS or NOHSS as the response variable. All statistical tests were two-tailed, with significance set at P < 0.05. Data analysis was conducted using R (version 4.3.3) and RStudio.

## Results

### Study participants

**Figure 2:**
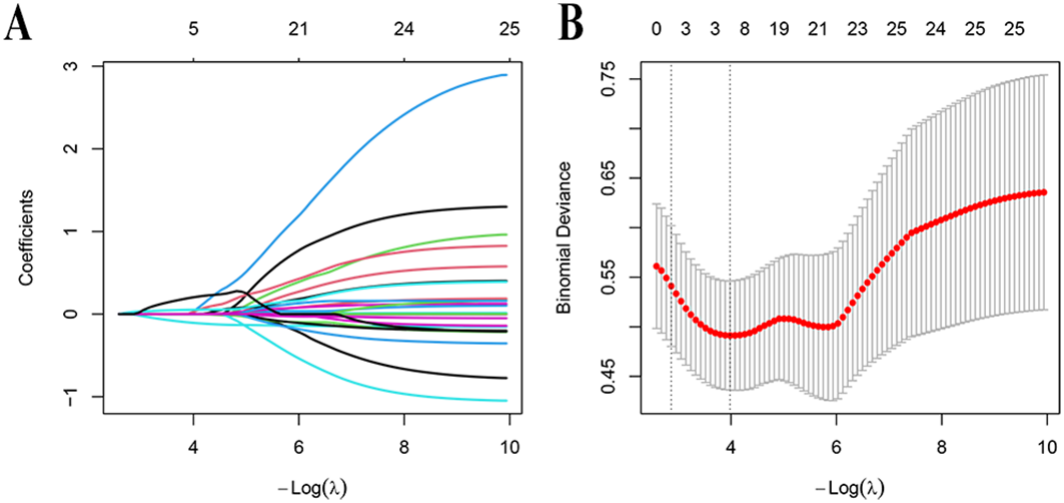
**(A)** Trajectory plot showing the selection process of variables from the dataset using LASSO regression for multivariate analysis; **(B)** Confidence intervals for each lambda value during LASSO regression.

This study enrolled 500 infertility patients who met the inclusion and exclusion criteria. Data on 6 clinical features (type of infertility, etiology of infertility, age, infertility duration, previous IVF cycles, and BMI) and 19 laboratory parameters were collected. The missing rate for all variables was below 5%, with missing data addressed through multiple imputations. The dataset was randomly split into training (n=350) and test (n=150) sets (**Figure 1**). Univariate logistic regression was first applied to the 25 variables within the training set. Baseline characteristics of the training set were compared between OHSS and NOHSS groups (**Table 1**), revealing no statistically significant differences (P > 0.05) in type of infertility, infertility duration, previous IVF cycles, or BMI. However, significant differences (P < 0.05) were observed in 2 clinical features (age and infertility duration), and 9 laboratory parameters (baseline FSH level, ratio of baseline LH/FSH, AMH level, FSH level on the first day of ovulation induction, P and E_2_ level on trigger day, number of follicles diameter ≥ 14mm, oocytes retrieved, and mature oocytes) between OHSS and NOHSS patients. Variables with P < 0.05 were retained as candidate set A (11 features). Separately, LASSO regression was applied to the same 25 variables, strictly within the training set. The penalty parameter λ was tuned with an inner ten-fold cross-validation loop, and the value that yielded the minimum mean cross-validated deviance (λ =1.1883757) was retained. At this λ, only four coefficients remained non-zero: Age, P levels on the trigger day, number of oocytes retrieved, and ratio of baseline LH/FSH, forming candidate set B. The shrinkage profiles of all 25 variables are displayed in **Figures 2A, B**, where the vertical line marks the chosen λ. To select the minimal set of features for OHSS, the final set of features for model construction was taken as the intersection of set A (univariate-selected) and set B (LASSO-selected). The final set of predictors for model construction was defined as the intersection of set A and set B, which resulted in the same four variables. These were subsequently carried forward for final model fitting and evaluation.

**Table 1:** Characteristics of the patients in training set.

Variables	Overall	NOHSS	OHSS	P value
		*N = 325*	*N = 25*	
Type of infertility:				0.423
Primary	174 (49.7%)	164 (50.5%)	10 (40.0%)	
Secondary	176 (50.3%)	161 (49.5%)	15 (60.0%)	
Etiology of infertility:				0.767
Female factor	152 (43.4%)	141 (43.4%)	11 (44.0%)	
Combined male and female factors	37 (10.6%)	33 (10.2%)	4 (16.0%	
Male factor	154 (44.0%)	144 (44.3%)	10 (40.0%)	
Other causes	7 (2.00%)	7 (2.15%)	0 (0.00%)	
Ovarian stimulation protocol:				0.369
Natural-cycle	1 (0.29%)	1 (0.31%)	0 (0.00%)	
Mild stimulation protocol using clomiphene citrate or letrozole [CC/LE mild stimulation]	14 (4.00%)	14 (4.31%)	0 (0.00%)	
Progestin-primed ovarian stimulation [PPOS] protocol	33 (9.43%)	33 (10.2%)	0 (0.00%)	
Antagonist protocol	261 (74.6%)	240 (73.8%)	21 (84.0%)	
Luteal Phase Ovulation Induction	6 (1.71%)	6 (1.85%)	0 (0.00%)	
Luteal Phase Short-Acting Long Protocol	1 (0.29%)	1 (0.31%)	0 (0.00%)	
Luteal Phase Modified Long Protocol	7 (2.00%)	7 (2.15%)	0 (0.00%)	
Follicular-phase long protocol	27 (7.71%)	23 (7.08%)	4 (16.0%)	
Group:				0.432
GnRH trigger	9 (2.57%)	9 (2.77%)	0 (0.00%)	
hCG trigger	45 (12.9%)	40 (12.3%)	5 (20.0%)	
dual trigger	296 (84.6%)	276 (84.9%)	20 (80.0%)	
Age [year]	33.0 [29.0;36.0]	33.0 [30.0;37.0]	28.0 [27.0;31.0]	<0.001
Infertility Duration [year]	3.00 [1.00;5.00]	3.00 [1.00;5.00]	2.00 [1.00;3.00]	0.029
Previous IVF cycles	0.00 [0.00;0.00]	0.00 [0.00;0.00]	0.00 [0.00;0.00]	0.552
BMI [kg/m^2^]	21.6 [20.0;24.1]	21.6 [20.0;24.0]	21.9 [21.1;25.7]	0.201
Baseline FSH [mIU/mL]	6.57 [5.19;8.30]	6.62 [5.37;8.43]	5.36 [4.06;7.25]	0.003
Baseline LH [mIU/mL]	4.38 [3.06;6.03]	4.35 [3.06;5.97]	4.54 [2.87;8.23]	0.208
Baseline E_2_ [pg/mL]	32.0 [24.0;44.0]	32.0 [24.0;44.0]	30.0 [26.0;35.4]	0.784
Ratio of baseline LH/FSH	0.63 [0.45;0.88]	0.61 [0.44;0.85]	0.92 [0.73;1.43]	0.001
AMH [ng/mL]	2.80 [1.34;5.08]	2.57 [1.27;4.75]	5.80 [4.67;7.35]	<0.001
Duration of stimulation [days]	10.0 [9.00;11.0]	10.0 [9.00;11.0]	10.0 [9.00;11.0]	0.245
Total dosage of Gn [IU]	1950 [1575;2325]	1950 [1575;2325]	1975 [1500;2400]	0.714
Hormone levels on the first day of ovulation induction				
FSH [mIU/mL]	6.24 [4.68;7.90]	6.30 [4.72;8.13]	5.48 [3.94;7.13]	0.034
LH [mIU/mL]	3.93 [2.74;5.44]	3.90 [2.76;5.36]	4.49 [2.52;8.23]	0.406
P [ng/mL]	0.46 [0.30;0.70]	0.46 [0.31;0.71]	0.42 [0.23;0.62]	0.259
E_2_ [pg/mL]	30.0 [22.0;41.0]	30.8 [22.0;43.0]	26.9 [21.0;35.0]	0.240
Hormone levels on trigger day				
LH [mIU/mL]	2.33 [1.39;3.69]	2.35 [1.45;3.69]	2.25 [1.05;3.32]	0.230
P [ng/mL]	0.86 [0.55;1.31]	0.85 [0.54;1.29]	0.96 [0.80;1.80]	0.014
E_2_ [pg/mL]	2270 [1207;3735]	2146 [1160;3557]	3843 [2901;5411]	<0.001
Number of follicles diameter ≥14mm	10.0 [5.00;14.0]	10.0 [5.00;14.0]	15.0 [12.0;21.0]	<0.001
Number of oocytes retrieved	9.50 [5.00;15.0]	9.00 [5.00;15.0]	19.0 [14.0;24.0]	<0.001
Number of mature oocytes	8.00 [4.00;14.0]	8.00 [4.00;13.0]	17.0 [11.0;20.0]	<0.001

### Model performance comparisons

The discriminative ability of the models was assessed using the C-index. All three models demonstrated acceptable discriminative performance (C-index > 0.80). Using bootstrap resampling with 1000 iterations, the optimism-corrected C-index was 0.90 (95% CI 0.81-0.97) (**Supplementary Figure S1B**) for the Random Forest model, 0.88 (95% CI 0.76-0.97) for the Xgboost model (**Supplementary Figure S1C**), and 0.81 (95% CI 0.68-0.91) for the Logistic model (**Supplementary Figure S1A**).The RandomForest model demonstrated the highest discrimination.

**Figure 3:**
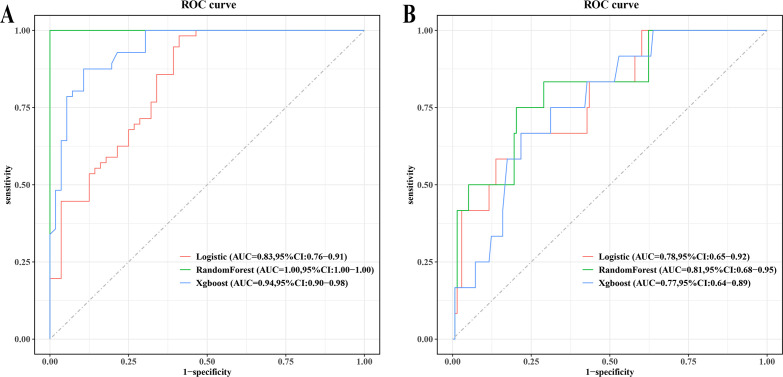
**(A)** ROC curves for each model in the training set; **(B)** ROC curves for each model in the test set. In the image, Logistic, logistic regression; RandomForest; Xgboost, extreme gradient boosting; ROC, receiver operating characteristic; AUC, area under the curve.

On the training set (**Figure 3A**), Random Forest achieved perfect AUC score of 1.00 (95% CI: 1.00-1.00). Xgboost also performed excellently with AUC of 0.94 (95% CI: 0.90-0.98). They were followed by Logistic with AUC of (95% CI: 0.76-0.91)). On the test set (**Figure 3B**), model discrimination was as follows: Random Forest, AUC 0.81 (95% CI: 0.68-0.95); Logistic, AUC 0.78 (95% CI: 0.65-0.92); and XGBoost, AUC 0.77 (95% CI: 0.64-0.89).

The observed overfitting phenomenon can be attributed primarily to class imbalance within the dataset, where the minority class (OHSS) was significantly underrepresented compared to the majority class (NOHSS). To address this issue, the SMOTE was applied exclusively to the training set to generate synthetic samples of the minority class and balance the class distribution. However, while SMOTE effectively mitigated imbalance-related bias during model training, it concurrently introduced artificial data points that amplified model complexity and fostered over-optimistic performance estimates on the training set. This manifested as near-perfect apparent discrimination on the training set (AUC 0.83-1.00 across models). However, the optimism-corrected C-index from bootstrap resampling (0.81-0.90) revealed substantially lower performance. This discrepancy indicates that the apparent AUC was inflated by the SMOTE-augmented training distribution, whereas the bootstrap C-index, derived from out-of-bag original samples, provided a more realistic estimate of discriminative capacity. These findings underscore the critical importance of independent test set evaluation in ML model development, as training set metrics alone, including those derived from resampled data, proved insufficient to ascertain true predictive capability.

**Table 2** summarizes detailed metrics for both training and test sets. On the training set, Random Forest achieved perfect scores (1.00) across all metrics-accuracy, sensitivity, specificity, precision, and F1 score. Xgboost attained F1 score (0.88), along with an accuracy of 0.88, sensitivity of 0.88, specificity of 0.89, and precision of 0.89. Logistic reached 0.98 sensitivity, along with an accuracy of 0.79, specificity of 0.59, precision of 0.71, and F1 score of 0.82. In the unseen test set, performance varied among models. RandomForest attained the highest F1 score (0.37), along with an accuracy of 0.79, sensitivity of 0.75, specificity of 0.80, and precision of 0.24.Xgboost and Logistic reached 0.67 sensitivity, along with an accuracy of 0.77, specificity of 0.67, precision of 0.21, and F1 score of 0.32.

**Table 2:** Comparison of each model’s performance in terms of accuracy, sensitivity, specificity, precision and F1 in the training set and test set.

Training set	Threshold	Accuracy	Sensitivity	Specificity	Precision	F1 score
Logistic	0.34	0.79	0.98	0.59	0.71	0.82
RandomForest	0.50	1.00	1.00	1.00	1.00	1.00
Xgboost	0.50	0.88	0.88	0.89	0.89	0.88
Test set	Threshold	Accuracy	Sensitivity	Specificity	Precision	F1
Logistic	0.57	0.77	0.67	0.78	0.21	0.32
RandomForest	0.45	0.79	0.75	0.80	0.24	0.37
Xgboost	0.50	0.77	0.67	0.78	0.21	0.32

**Figure 4:**
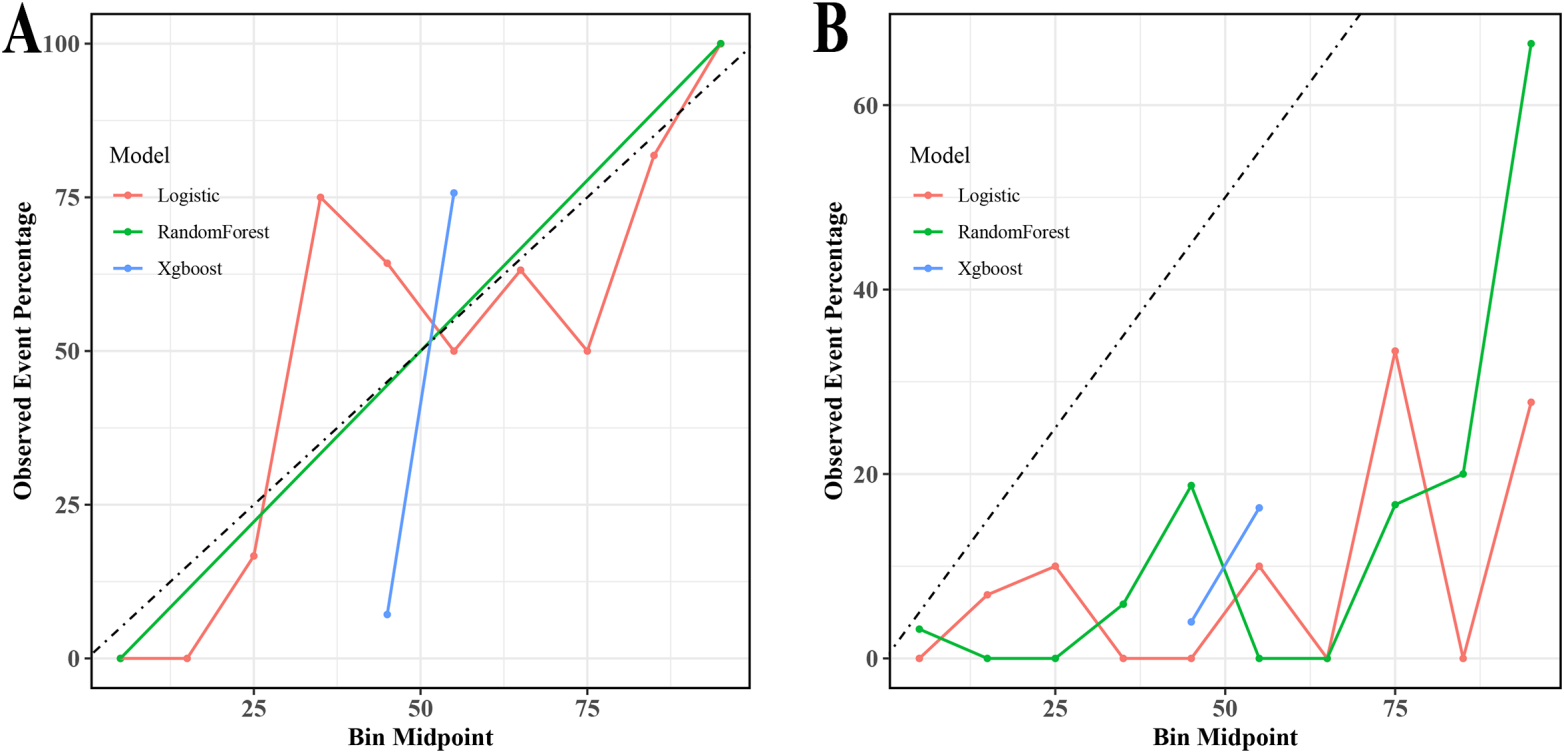
**(A)** Calibration curves for each model in the training set; **(B)** Calibration curves for each model in the test set.

**Figure 5:**
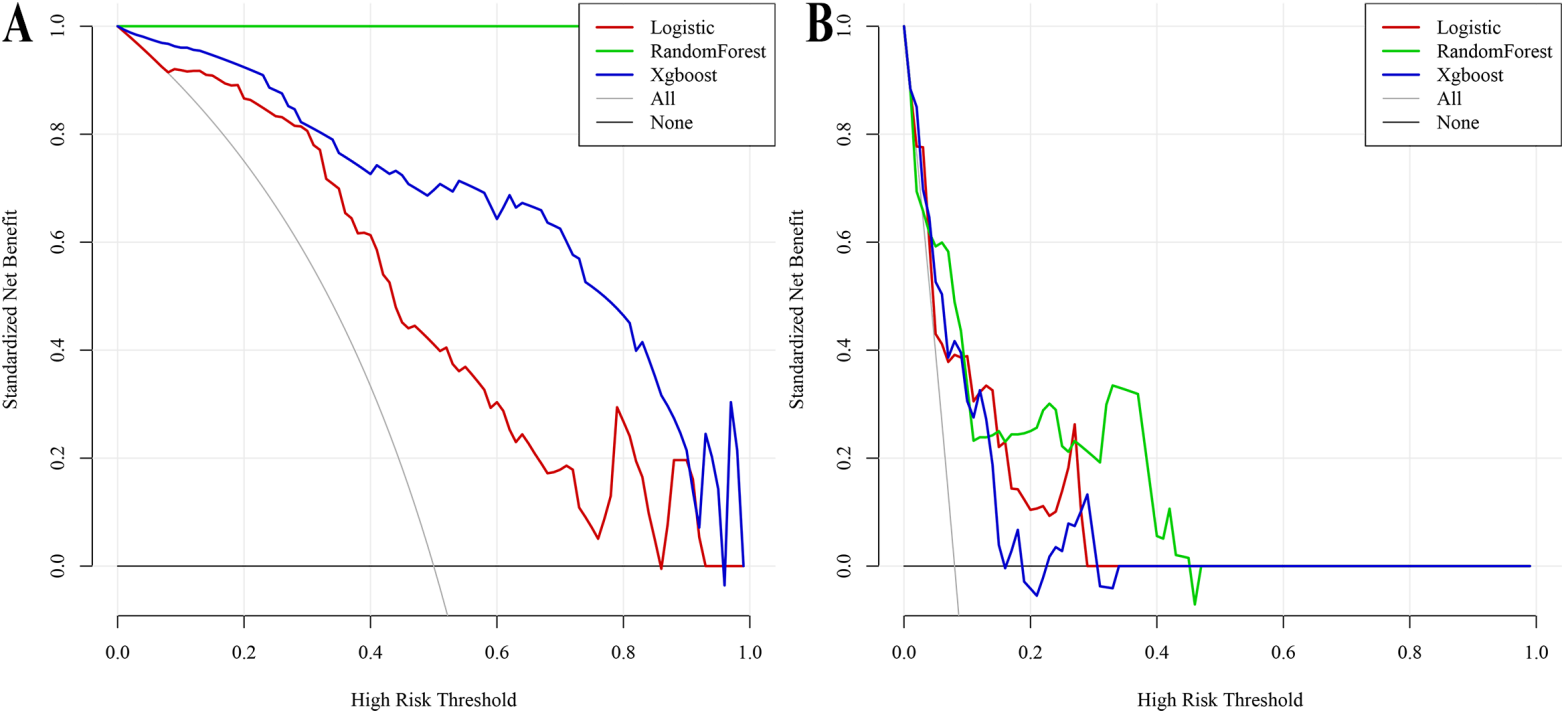
**(A)** DCA, Decision curve analysis curves for each model in the training set; **(B)** DCA curves for each model in the test set.

On the training set, Random Forest model exhibited relatively smooth curves close to the diagonal line. In the low predicted probability range, the Random Forest and Logistic models performed reasonably well. However, on the test set, all models deviated significantly from the diagonal, indicating a substantial deterioration in calibration performance. In the low predicted probability range (0-25%), the Random Forest model performed relatively well (**Figures 4A, B**). In terms of clinical applicability, the training set DCA curves demonstrated strong net benefit; however, performance on the test set was more limited (**Figures 5A, B**). Despite this, within the clinically relevant risk threshold range of 1-10%, where OHSS incidence is most concentrated, the RandomForest model exhibited competitive and robust net benefit, making them the most suitable choices for post-retrieval OHSS risk stratification (**Figure 5B**).

### Bootstrap feature selection stability analysis

Bootstrap feature selection stability analysis (1000 iterations) revealed heterogeneous reliability among the candidate predictors. The number of oocytes retrieved demonstrated the highest stability (mean rank 1.84 ± 0.99, selection frequency 0.914), retained in the top three across 91.4% of resamples. Ratio of baseline LH/FSH showed high stability (mean rank 2.29 ± 1.08, selection frequency 0.819). Age showed moderate stability (mean rank 2.69 ± 1.09, selection frequency 0.695), while P levels on the trigger day exhibited low stability (mean rank 3.18 ± 0.84, selection frequency 0.572), with rankings varying substantially across bootstrap samples (**Table 3**). These findings indicate that the predictive signal is concentrated in a limited subset of baseline variables, with the number of oocytes retrieved and ratio of baseline LH/FSH representing the most robust and reproducible predictors of post-retrieval OHSS risk stratification.

**Table 3:** Bootstrap feature selection stability analysis (n=1000 iterations).

Feature	Mean rank	Rank SD	Selection freq	Stability grade	Rank CI	Mean importance
Number of oocytes retrieved	1.84	0.99	0.914	High	1.84 ± 0.99	0.0234
Ratio of baseline LH/FSH	2.29	1.08	0.819	High	2.29 ± 1.08	0.0198
Age	2.69	1.09	0.695	Moderate	2.69 ± 1.09	0.0169
P levels on the trigger day	3.18	0.84	0.572	Low	3.18 ± 0.84	0.0135

### Interpretability analysis

**Figure 6:**
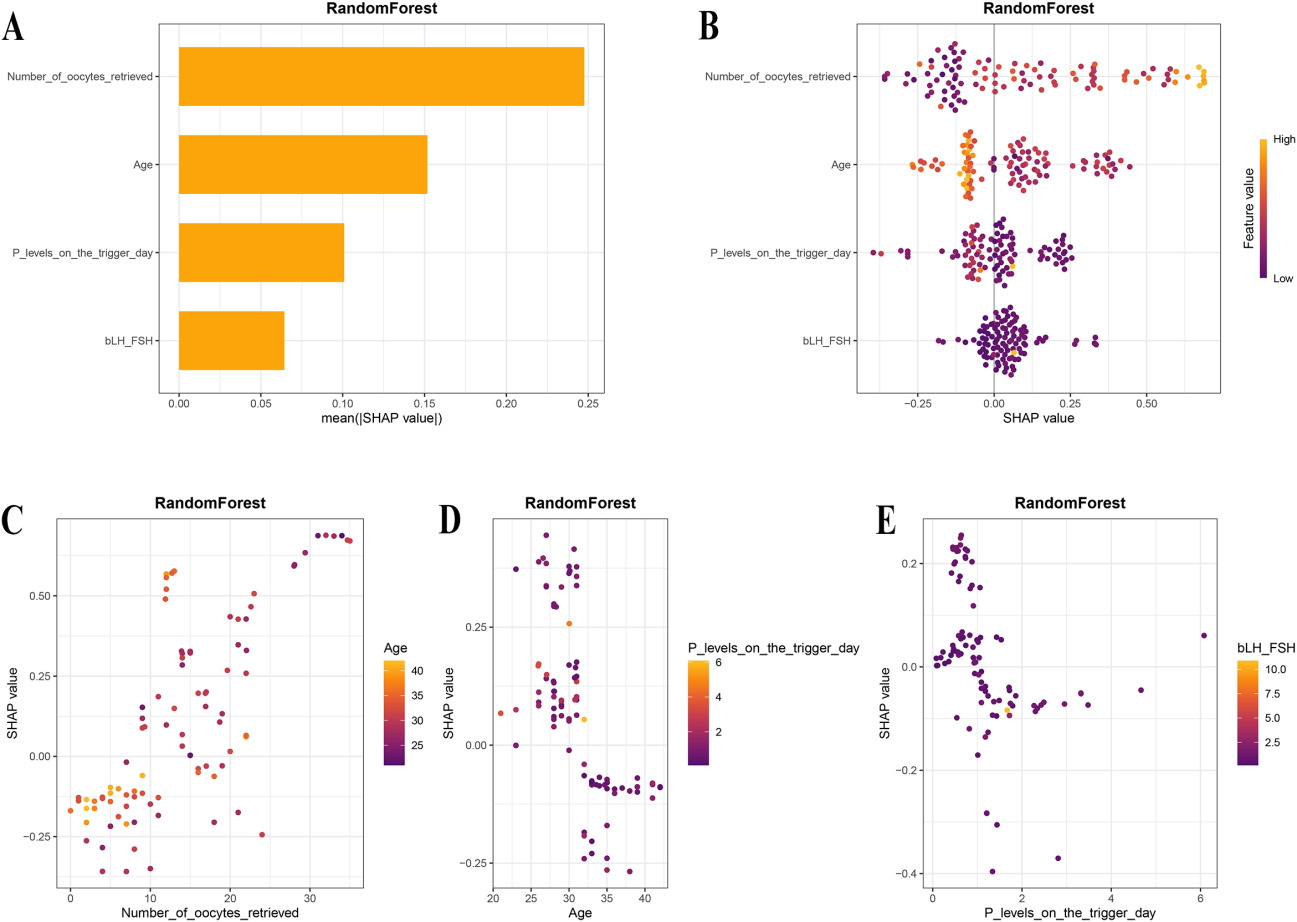
Interpretability analysis of RandomForest model. **(A)** Importance ranking plot of features of the RandomForest model. **(B)** SHAP dendrogram of features of the RandomForest model. **(C–E)** Scatter plots of SHAP values for Top 3 features.

In order to visually explain the selected variables, we used SHAP to illustrate how these variables affect the OHSS risk in the RandomForest model. **Figure 6A** shows the top 4 risk variables evaluated by the average absolute SHAP value. Higher SHAP value indicates greater contribution to the OHSS risk. **Figure 6B** uses a bee swarm plot to show how each feature influences the model’s predictions. The points are color-coded, with purple representing low risk and yellow representing high risk, making the relationship between a feature’s value and its impact easy to see. Among all features, number of oocytes retrieved has the strongest influence. Higher values of this feature (shown in yellow) correspond to a strong positive effect on the prediction, meaning they are associated with higher OHSS risk. Age is another important feature, where a higher value (older age) generally lowers the predicted risk. P levels on the trigger day also contribute to the model’s output. **Figures 6C–E** provided another view with scatter plots, confirming these relationships. An increase in number of oocytes retrieved significantly raises the OHSS risk. In contrast, age shows a negative correlation with the prediction. P levels on the trigger day demonstrates a non-linear relationship with OHSS risk, where low P levels (< 1.0 ng/mL) exhibit the highest positive SHAP values as strong risk predictors, while extremely elevated P (>2.0 ng/mL) paradoxically demonstrates negative contributions. This analysis helps clarify the role of each feature under different conditions, improving the model’s interpretability and providing clinical insights for OHSS risk assessment.

**Figure 7:**
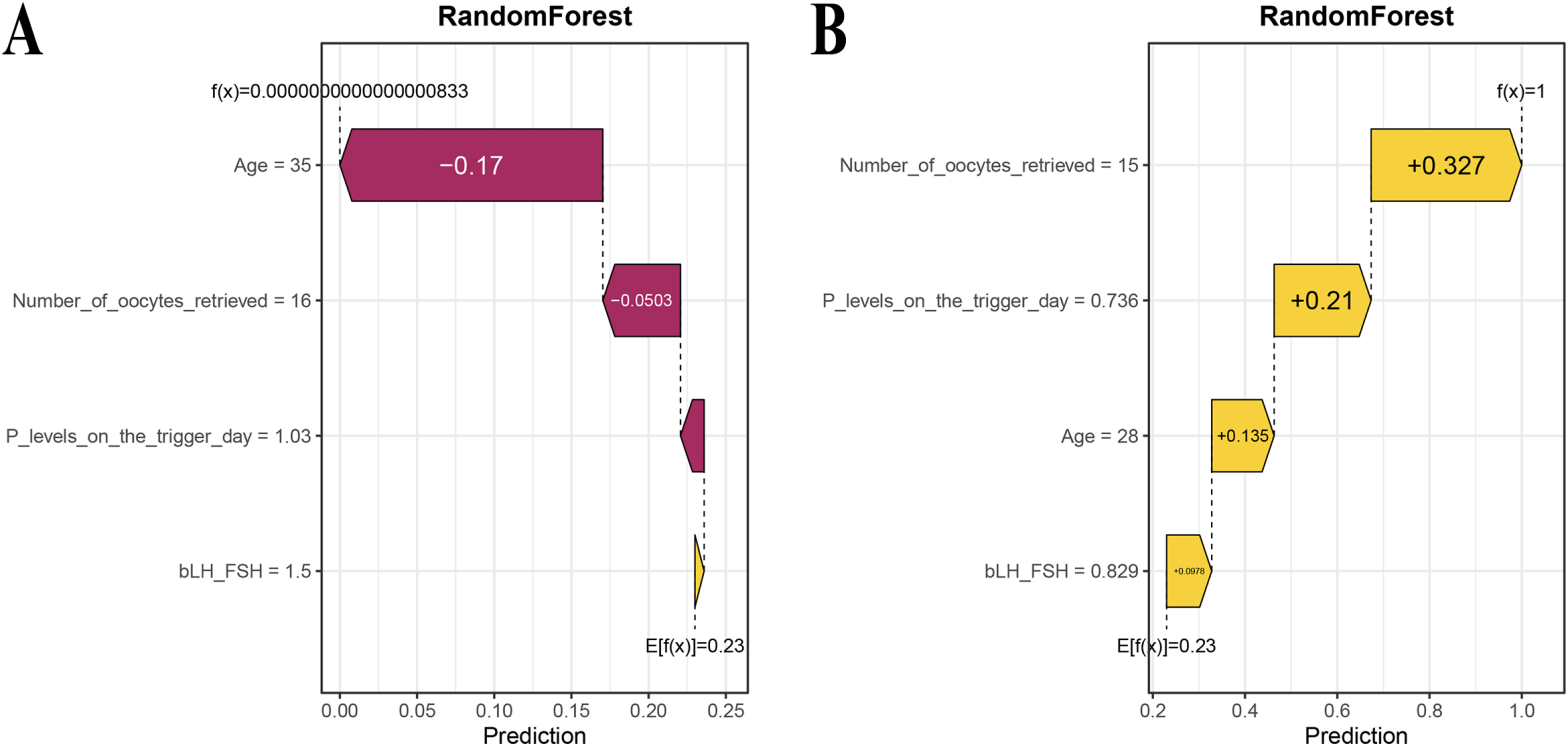
Local model explanation by the SHAP method. **(A)** Waterfall plot for one NOHSS patient. **(B)** Waterfall plot for one OHSS patient.

**Figure 7A**, B are waterfall plots that break down the prediction for a single patient. Each bar represents a feature’s contribution: purple for variables that decrease risk and yellow for those that increase it. In the low-risk patient example (**Figure 7A**), the model identified age of 35 years (SHAP value: -0.11) as the principal variables reduce OHSS risk. The final prediction score of 0.00 was significantly lower than the mean prediction value of 0.23 indicating low OHSS risk. Conversely, in the high-risk patient example (**Figure 7B**), number of oocytes retrieved of 15 emerged as the predominant risk-enhancing factor (SHAP value: +0.327). Additional contributors included P levels on the trigger day of 0.736 pg/mL (SHAP value: +0.21) and younger age of 28 years (SHAP value: +0.135). The prediction score of 1.00 significantly higher than the mean value, indicating high OHSS risk.

## Discussion

This study exploratorily developed and internally validated three ML models for post-retrieval OHSS risk stratification in patients undergoing ART treatment. In the test set, the RandomForest model demonstrated optimal discriminatory performance, achieving an AUC of 0.81, sensitivity of 0.75, and specificity of 0.80. Furthermore, LASSO and SHAP analysis enhanced model transparency by identifying the number of oocytes retrieved, age, P levels on the trigger day, and ratio of baseline LH/FSH as the most influential predictors, thereby providing clinically interpretable insights.

Notably, the RandomForest model achieved 0.80 sensitivity, indicating that the vast majority of patients who actually developed OHSS were correctly identified, with minimal proportion of missed evaluation. This is particularly crucial in the clinical context of OHSS risk stratification, where even a small number of false negatives may result in high-risk patients not receiving necessary preventive measures. Although specificity was relatively lower (0.75), implying that some low-risk patients may be over-warned, such over-warning carries substantially lower clinical costs compared to missed evaluation in practice. The importance of accurate OHSS risk stratification is further underscored by its significant impact on pregnancy outcomes. A 2021 meta-analysis demonstrated that OHSS patients experience adverse pregnancy outcomes 8.8 more frequently than NOHSS patients (30). Consistent with these findings, previous studies have also reported miscarriage rates of 12.2-29.8% in OHSS-affected pregnancies (7, 31, 32), as well as higher rates of pre-eclampsia and preterm birth relative to NOHSS IVF controls (32, 33). Through further clinical assessment and stratified management (such as cycle segmentation, GnRH antagonist protocol modifications, or low-dose hCG triggers), these false-positive patients can be reclassified without delaying treatment or causing substantial harm (34).

SHAP analysis revealed that the number of oocytes retrieved, age, and P levels on the trigger day were the three most important features for predicting OHSS risk at the population level (35–37). This finding is highly consistent with existing literature and pathophysiological mechanisms. The number of oocytes retrieved directly reflects ovarian response to gonadotropin stimulation and serves as the most direct indicator of OHSS risk; large numbers of retrieved oocytes lead to significant ovarian enlargement and substantial secretion of vasoactive substances such as VEGF, thereby increasing vascular permeability-the central pathogenic mechanism of OHSS (15, 38). Age functions as a negative predictor, closely related to ovarian reserve and responsiveness-younger patients typically possess better ovarian reserve and more sensitive responses to stimulation, thus carrying higher OHSS risk (37). P levels on the trigger day, as a biomarker of follicular luteinization and ovarian hyper-response, predict extensive granulosa cell maturation and premature progesterone elevation at high levels, positively correlating with OHSS risk (39). However, our research showed that the combination of extremely low P (< 1.0 ng/mL) constitutes the highest-risk phenotype, whereas supraphysiological P levels (>2.0 ng/mL) paradoxically lose predictive value, suggesting an “optimal interval” effect for P-level-based OHSS risk stratification. Beyond population level interpretations, SHAP analysis enables individualized risk stratification by revealing how feature contributions vary across patients. For example, **Figure 7** illustrates two contrasting cases: Patient A exhibited minimal SHAP values for oocyte-related variables, with age driving the prediction instead, whereas Patient B showed dominant contributions from retrieved oocyte counts. This heterogeneity demonstrates that the model does not apply uniform rules to all patients but dynamically adjusts its predictive logic based on individual feature profiles. Such capability is clinically valuable for identifying patient specific risk patterns. Thus, SHAP-enhanced interpretability transforms the model from a “black box” predictor into a clinically actionable tool for personalized OHSS prevention strategies.

This study possesses several key strengths. First, the comprehensive comparison of three ML algorithms identified RandomForest as particularly effective for this risk stratification. Second, the integration of SHAP addresses the critical barrier of model interpretability, rendering model logic accessible to clinicians while enabling intuitive understanding of each patient’s risk composition. For instance, young patients with high AMH and number of oocyte retrieved has higher risk scores with clear factor contributions, whereas older patients with diminished ovarian reserve maintain relatively low predicted risk despite higher gonadotropin doses - facilitating transparent risk communication and collaborative decision-making. Third, the use of readily available clinical variables enhances generalizability to routine practice, these tools might eventually serve as hypothesis-generating frameworks to inform-but not replace-clinical judgment in post-retrieval risk stratification, potentially informing consideration of preventive interventions (e.g., cancellation of fresh embryo transfer, additional use of GnRH antagonists, albumin supplementation, or deferred embryo transfer) based on actual oocyte numbers.

This study has several limitations. First, the relatively low OHSS incidence (8%) resulted in class imbalance, which may have influenced model calibration and contributed to the modest F1 score (0.37). While our random oversampling during training partially mitigated this, future studies should consider advanced resampling techniques or synthetic data generation. Second, the small sample size, particularly with only 40 OHSS-positive cases, substantially limited model stability and generalizability. Future studies should prioritize multi-center recruitment to achieve adequate sample size and event counts. Third, this study employed a single-center, retrospective design, susceptible to selection and information bias. The lack of external validation means that our findings may not generalize to other settings. Future work must prioritize external validation using the following concrete strategies: (1) temporal validation using data from a later period at the same center to assess model stability over time; (2) geographic validation across two or more independent centers with different patient demographics and IVF protocols; and (3) prospective validation in a separate cohort to evaluate real-world performance. Only after successfully passing these external validation steps could the model be considered for clinical implementation. Fourth, model specificity requires improvement; future work may reduce false-positive rates and enhance prediction precision through feature engineering (incorporating additional biomarkers, ultrasound parameters) and algorithm optimization (cost-sensitive learning, threshold adjustment). Moreover, there remains a gap between prediction and clinical action: the model can identify high-risk patients, but how to translate this prediction into specific preventive interventions (e.g., individualized gonadotropin dosing, cycle cancellation, or elective freeze-all) requires further implementation research.

Furthermore, this study did not assess intervention effectiveness, whether clinical decision adjustments based on model predictions can actually reduce OHSS incidence and improve patient outcomes. Conducting randomized controlled trials comparing ML-guided individualized protocols versus standard protocols will be a critical step in validating the clinical value of these models.

## Conclusion

In summary, the RandomForest-based ML model developed in this study stratify OHSS risk in ART patients using routinely collected clinical data, demonstrating high sensitivity and good discriminatory capacity. The integration of SHAP analysis provides explicit, individualized risk interpretation, helping clinicians intuitively understand the impact of key predictive features such as number of oocytes retrieved, age, and P levels on the trigger day. This tool shows promise as an important component of risk stratification support systems, identifying high-risk patients, generating hypotheses for future research. Ultimately, it may help reduce the incidence and severity of OHSS, thereby improving the safety and efficiency of ART treatment, although these findings are exploratory, prospective external validation remains warranted before any clinical application.

## Data Availability

The original contributions presented in the study are included in the article/**Supplementary Material**. Further inquiries can be directed to the corresponding author.
