# Supplementary material for: Development and internal validation of a post-retrieval machine learning models for OHSS risk stratification in assisted reproductive technology: an exploratory study
Source: Front Endocrinol (Lausanne). 2026 May 25;17:1826843. doi: 10.3389/fendo.2026.1826843 (PMC13243076; doi:10.3389/fendo.2026.1826843)
Supplement: Supplementary file 1 [file SupplementaryFile1.docx]

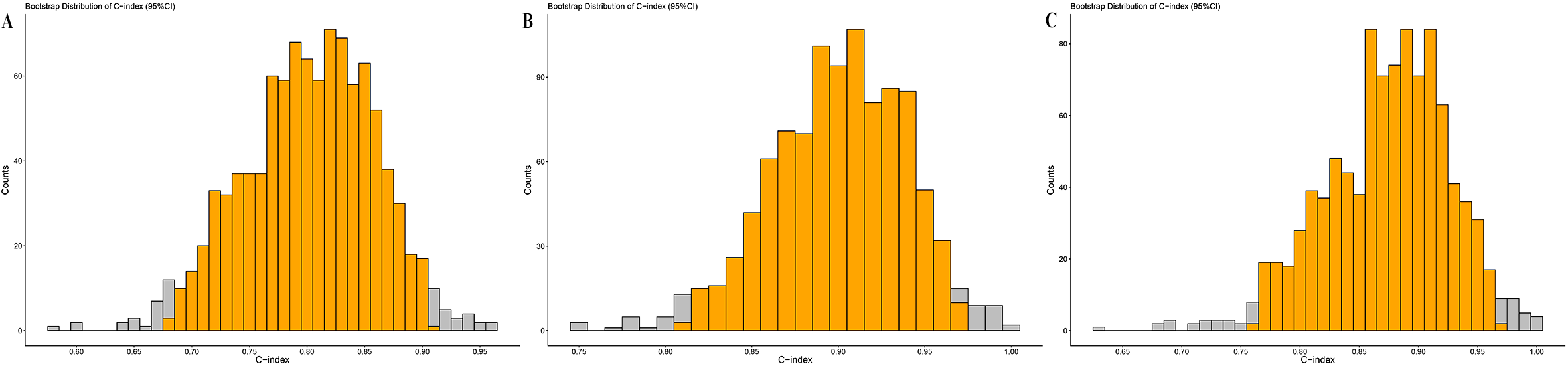


Figure S1 Distribution of C-index values for three machine learning models based on 1000 bootstrap resampling iterations. (A) Logistic: logistic regressiont; (B) RandomForest; (C)Xgboost: extreme gradient boosting.
